# Mapping urban-rural gradients of settlements and vegetation at national scale using Sentinel-2 spectral-temporal metrics and regression-based unmixing with synthetic training data

**DOI:** 10.1016/j.rse.2020.111810

**Published:** 2020-09-01

**Authors:** Franz Schug, David Frantz, Akpona Okujeni, Sebastian van der Linden, Patrick Hostert

**Affiliations:** a Earth Observation Lab, Geography Department, Humboldt-Universität zu Berlin, Unter den Linden 6, 10099 Berlin, Germany; b Institut für Geographie und Geologie, Universität Greifswald, Friedrich-Ludwig-Jahn-Str. 16, 17487 Greifswald, Germany; c Integrative Research Institute on Transformations of Human-Environment Systems (IRI THESys), Humboldt-Universität zu Berlin, Unter den Linden 6, 10099 Berlin, Germany

**Keywords:** Built-up, Urban, Settlement, Land cover, Germany, Austria, Unmixing, Regression

## Abstract

The increasing impact of humans on land and ongoing global population growth requires an improved understanding of land cover (LC) and land use (LU) processes related to settlements. The heterogeneity of built-up areas and infrastructures as well as the importance of not only mapping, but also characterizing anthropogenic structures suggests using a sub-pixel mapping approach for analysing related LC from space. We implement a regression-based unmixing approach for mapping *built-up surfaces and infrastructure*, *woody vegetation* and *non-woody vegetation* for all of Germany and Austria at 10 m resolution to demonstrate the potential of sub-pixel mapping. We map LC fractions for one point in time, using all available Sentinel-2 data from 2017 and 2018 (<70% cloud cover). We combine the concept of synthetically mixed training data with statistical aggregations from spectral-temporal metrics (STM) derived from Sentinel-2 reflectance time series. We specifically examine how STM can be used for creating synthetically mixed training data. STM are known to facilitate large area mapping by being largely independent of image acquisition dates and inherently incorporate phenological information. Vegetation is an important part of settlements and time series information supports its mapping. Synthetically mixed training data facilitates a streamlined training by using pure reference spectra to generate artificial mixtures as input to regression modelling of LC fractions in mixed pixels. We here show how combining both offers great potential for wall-to-wall LC fraction mapping. We further investigate the positive effect of STM on map results by comparing the performance of different subsets of STM combinations. Our results indicate that many STM combinations containing spectral variability and vegetation indices provide suitable input to creating synthetic training data for regression-based fraction mapping. Results for *built-up surfaces and infrastructure* (MAE 0.13/RMSE 0.18 at 20 m resolution), *woody vegetation* (0.18, 0.22) and *non-woody vegetation* (0.14, 0.19) are highly consistent across Germany and Austria. Only a few surface types were not accurately predicted in our nation-wide mapping. Further research is required to optimize mapping of temporally invariant bare soil and rock surfaces that show spectral similarity to built-up surfaces and infrastructure. The proposed methodology combines benefits of both regression-based modelling with synthetically mixed training data and STM, and thus facilitates mapping of LC fractions on a national scale and at high resolution. Such information will allow to better characterize settlements and identifying processes such as densification that are best represented by continuous LC mapping.

## Highlights

• Sub-pixel built-up, woody & non-woody vegetation fractions mapped at national scale.
• Wide range of rural and urban settings investigated for Germany and Austria.
• Synthetic training data from spectral-temporal metrics leads to robust results.
• A single model achieved MAE of 13–18% (RMSE 18–22%) from coastal to alpine regions.
• Sub-pixel fractions structurally characterize settlements in Germany and Austria.

## 1 Introduction

Human activity has shaped land cover (LC) and land use (LU) dynamics over the last decades (Lambin and Geist, 2006). Song et al. (2018) found that about 60% of all global (and up to 86% of all European) LC and LU change from 1982 to 2018 can be directly related to human activities. Processes include forestry and agriculture, but also settlement and infrastructure development. Despite their relatively small overall share of LC, settlements have an important effect on LC and LU dynamics within and outside of city boundaries, locally and globally (Foley et al., 2005; Turner et al., 2007). Rapid population growth - world population is predicted to grow from 7.4 billion in 2015 to 9.2 billion in 2040 (UNDESA, 2019) - suggests that understanding spatial-temporal patterns of settlement dynamics at national scales is essential from the perspective of planning and service provision, climate change adaptation, LU management and related aspects also addressed in the Sustainable Development Goal 11 on “Sustainable Cities and Communities” (United Nations, 2018).

Even though more people are expected to live in cities (64.5% in 2040 as compared to 53.9% in 2015 (UNDESA, 2018, UNDESA, 2019)), population growth will not only occur in urban agglomerations, but also in smaller urban centers, their surroundings and less populated areas in the countryside (Melchiorri et al., 2018). Despite lower population densities, those areas account for a substantial amount of infrastructure, especially with regard to transportation. For example, emissions from transportation greatly depend on the patterns of settlement development (Baiocchi et al., 2015). Within settlements, the type and structure of sub-areas also has a large impact on different aspects of human life quality, mobility behaviour, settlement resilience and environmental impacts of built-up features (Stokes and Seto, 2019). This is why settlement mapping requires a spatially comprehensive approach encompassing the entire urban-rural gradient, where the character regarding building density, building structure, object sizes, green space and infrastructure varies gradually. It is, thus, also essential to capture LC distribution in and between settlements over large areas. Particularly with regard to sub-pixel structures, discrete LC mapping approaches are an over-simplification of the human environment (Small and Sousa, 2016). This is specifically challenging when mapping infrastructure and the LC composition of smaller settlements and in rural areas. Even though increased spatial resolution can partly counter this problem, van der Linden et al. (2018) found that at 9 m spatial resolution, 80% of the pixels still contained two or more surface types. Moreover, mapping the type and characteristics of settlements also requires knowledge about less abundant LC components, including different vegetation types (Wentz et al., 2018). The dominance of highly diverse mixtures in pixels therefore renders quantitative sub-pixel mapping a favourable approach for consistently characterizing urban-rural gradients (MacLachlan et al., 2017).

Large area classification approaches have been established for settlement mapping using different classification schemes, data types and semantics. Global and continental products provide ready-to-use settlement extent maps. The *Global Urban Footprint* (GUF, Esch et al., 2013) and the *Global Human Settlement Layer* (GHSL, Pesaresi et al., 2013; Corbane et al., 2019) are among the best-known binary products based on remote sensing. An overview of discrete large area products is provided in Schneider et al. (2010), Klotz et al. (2016) and Small and Sousa (2016). Imperviousness is a continuous phenomenon that is often considered a proxy for settlement presence (Weng, 2012). LC classification products such as FROM-GLC10 (Gong et al., 2019) account for large area imperviousness and specific large area imperviousness products, such as in the US National LC Database (Homer et al., 2004), the *Copernicus Imperviousness Layer* (Langanke et al., 2018) or the *Global Man-made Impervious Surface* dataset (Brown de Colstoun et al., 2017) offer continental or global estimates for a single imperviousness class. LC fraction mapping has also been widely applied with a focus on vegetation, where it is often referred to as *vegetation continuous fields*. DeFries et al. (1999) mapped global continuous fields vegetation characteristics using MODIS imagery and linear mixture models, Asner et al. (2005) mapped fractional vegetation cover in the Amazon Basin using Landsat ETM+ data and global forest cover maps were produced using large amounts of MODIS and Landsat data (Hansen et al., 2003). Those approaches do not focus on settlement features and often made use of lower resolution imagery for global applications. The idea of LC fraction mapping is also represented in the vegetation - impervious surface - soil (V-I-S) model that was designed and locally used to characterize urban morphology based on spectral information (Ridd, 1995; Phinn et al., 2002). The potential of decameter resolution imagery for large area multi-class sub-pixel LC mapping is still unclear. *Spectral Mixture Analysis* (SMA, Roberts et al., 1993) and related methods such as *Multiple Endmember Spectral Mixture Analysis* (MESMA, Roberts et al., 1998) have been widely used for fraction mapping in various applications, including components of settlement LC (Wu and Murray, 2003; Powell et al., 2007; Yang and Li, 2015; Wetherley et al., 2018). So have regression-based approaches (Yuan et al., 2008; Kaspersen et al., 2015). However, many regression-based fraction mapping approaches require a complex training procedure, as they use training areas derived from very high resolution imagery, referred to as map-based training (Sexton et al., 2013).

Okujeni et al. (2013) introduced a regression-based unmixing approach using synthetically mixed training data. This approach utilizes image-based spectral signatures of known pure surface types (a *library*) and generates synthetic mixtures of those spectra in order to simulate continuous training data that can be used for regression training. Using library data to create artificial training data largely facilitates the training process. The approach was previously used to map an extended V-I-S scheme for settlement LC composition (Okujeni et al., 2015; Priem et al., 2019). The concept was extended through an ensemble approach (Okujeni et al., 2017) and showed good results in different local and regional environments (Okujeni et al., 2018; Schug et al., 2018; Suess et al., 2018). However, the approach has not been used to map two vegetation types and has never been used at national scales. Finally, training data has never been generated from STMs. The robustness towards regional spectral variability and seasonality was not yet studied.

Workflows based on single-date acquisitions or best-available pixel composites are limited when it comes to differentiating spectrally similar surface types. Whereas spectra of materials used for buildings and infrastructure (e.g. concrete) are distinct from the spectral signatures of vegetated pixels, they are physically and spectrally similar to some natural soil and bare rock surfaces (Stefanov et al., 2001; Herold et al., 2004). This can particularly lead to inconsistent estimates of different sub-pixel fractions in areas with phenological variation. For example, post-harvest open soil can be miss-interpreted as a built-up surface on agricultural fields. That challenge can be encountered by incorporating all available data within a study period and, thus, making use of information on vegetation phenology to overcome seasonal spectral ambiguities. Spectral-temporal metrics (STM) from image time series inherently include phenological information based on pixel-wise reflectance statistics or derived indices. Temporal Normalized Difference Vegetation Index (NDVI) metrics were, together with phenometrics or land surface temperature, shown to be useful to measure phenological variability (Reed et al., 1994) or to enhance LC change detection (Borak et al., 2000). NDVI metrics have also been used for regression-based unmixing with map-based reference spectra (Kaspersen et al., 2015). STM were shown to improve classification quality for discrete multi-class large area LC maps including seasonal and *artificial* surfaces (Pflugmacher et al., 2019). Schneider (2012) found that, depending on the classifier, STM from Landsat imagery increase discrete classification quality for change mapping of *urban areas* in Chinese cities. The seasonal aspect of the data was particularly useful for separating bare ground, seasonal post-harvest agriculture or temporary construction sites from permanent *urban surfaces*.

Freely available Sentinel-2 data from ESA's Copernicus programme offer the potential for mapping infrastructure and built-up features more precisely than comparable decameter resolution satellite missions: (1) the average five-day revisit rate of the dual Sentinel-2A/B constellation substantially improves data availability compared to missions like Landsat (16 days, Li and Roy, 2017) and (2) the spatial resolution of 10 m – 20 m (band-dependent) seems promising for mapping infrastructure and built-up features.

Our overarching goal is to map LC fractions for Germany and Austria to describe the LC composition of settlements and infrastructure consistently along urban-rural gradients. We use STM from all available Sentinel-2A/B imagery in 2017 and 2018 (<70% cloud cover) for regression-based unmixing with synthetically mixed training data. We map fractions of *built-up features and infrastructure* (including buildings, roads and man-made pervious features such as railway tracks, but excluding natural impervious features such as bare rock), *woody vegetation* (including deciduous forests, coniferous forests, woody shrubs and tree crops) and *non-woody vegetation* (including grassland and non-tree crop agriculture) for one point in time. We implement an automated workflow that is able to unmix LC at 10 m spatial resolution with a single set of training data for the entire area. We use per-pixel STM of analysis ready image time series to include phenology-driven spectral variation, to increase data comparability of different sub-regions and to better separate LC types susceptible to spectral-temporal confusion. We specifically address the following research objectives:

• We examine if the combined use of STM derived from Sentinel-2A/B time series and the concept of synthetically created training data allows for accurate fraction mapping of built-up surfaces and infrastructure, woody and non-woody vegetation with regression-based modelling across Germany and Austria.
• We compare the performance of different STM and STM combinations and their suitability for the generation of synthetic training data, and this way explore whether the advantageous temporal component of STM is preserved during synthetic mixing, knowing that STM do not represent actually measured surface spectra.
• We identify the value of quantitative land cover mapping for better understanding the type and structure of settlements on a nation-wide wall-to-wall level.

## 2 Study area and data

### 2.1 Study area

**Fig. 1:**
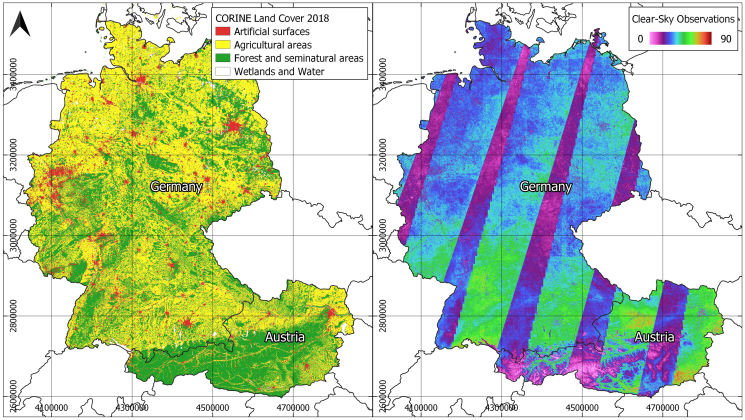
Left: CORINE Land Cover Product 2018. Right: Clear-sky observations per pixel in the study area. Stripes of higher availability are due to Sentinel-2A/B orbit overlaps. Total range of 0–110 observations was clipped at 90 for illusStrative purposes.

We mapped LC fractions of *built-up features and infrastructure*, *woody vegetation* and *non-woody vegetation* within the borders of Germany and Austria, covering a total area of about 440,000 km^2^ (Fig. 1) and providing very large gradients in the distribution of LC. Germany has a population of about 82.3 million, Austria of about 8.8 million, with a respective population density of 236.1 inhabitants per km^2^ in Germany and 106.2 in Austria (UNDESA, 2019). The proportion of people living in urban areas is 77.3% in Germany and 58.3% in Austria, with both high-density and rural settlements being abundant across the study area (United Nations, 2015). Among settlements, both *green* and *gray* areas can be found. According to the CORINE Land Cover product 2018 (EEA, 2007, EEA (Ed.), 2019), 9.4% of the total land surface in Germany (5.9% in Austria) is covered by *artificial areas*, showing the relevance of settlement mapping with regard to surface area. The most common LC types in both countries are *agricultural areas* (57.3% in Germany, 31.9% in Austria) and *forests* (31.6%/54.0%). The study area is located within the *Temperate Broad-Leaved and Mixed Forests* biome in most of Germany and the North-East of Austria and the *Temperate Conifer Forests* biome in the alpine regions (Olson et al., 2001). Settlement structure in central Europe, also represented in our study area, is highly diverse, ranging from medieval urban cores to homogeneous pre- and post-industrialization housing, low to medium density residential and commercial buildings built after the end of World War II, Soviet architecture and modern and post-modern international-style developments.

### 2.2 Data

We used all Sentinel-2A/B Multispectral Imager (MSI) Level 1C acquisitions with cloud coverage <70% for 2017 and 2018. Sentinel-2B became operational in mid-2017 (ESA Earth Online, 2017), and the constellation was fully ramped up on 17 February 2018 (ESA Sentinel Online, 2018), thus providing 5-day nadir revisit frequency thereafter. The study area is covered by 86 Sentinel-2A/B MGRS tiles, including orbit overlaps. 15,398 image acquisitions were processed to Analysis Ready Data (ARD) through the Framework for Operational Radiometric Correction for Environmental monitoring (FORCE, Frantz, 2019). The main processing steps included cloud and cloud shadow masking (Frantz et al., 2018), radiometric correction including corrections for atmospheric, topographic, BRDF, and adjacency effects (Frantz et al., 2016b), re-projection into a common coordinate system (EPSG: 3035) and creating data cubes for efficient analysis. FORCE provides the ten land-application bands as Bottom-of-Atmosphere reflectance with a resolution of 10 m. The initial 20 m bands are improved to 10 m using a multi-temporal data fusion approach (Frantz et al., 2016a). Up to 110 clear-sky observations per pixel (excluding cloud, cloud shadow, snow, saturated pixels as well as sub-zero reflectance values) were available within the study period, with an average observation count of about 34 (Fig. 1).

## 3 Methods

In a first step, we derived STM of reflectance and the NDVI from all imagery in the study period. In a second step, reference locations of pure LC types were identified. We systematically selected feature combinations that were used to respectively extract features at the reference locations. We created artificial mixtures from each library of STM combinations and trained individual regression models for each LC type. Those were used to predict LC fractions and were evaluated based on a stratified random validation scheme. A specific STM combination was eventually selected to create a model for each LC type for mapping fractions in the entire study area. Water, slope and snow masks were applied.

### 3.1 Spectral-temporal metrics

**Fig. 2:**
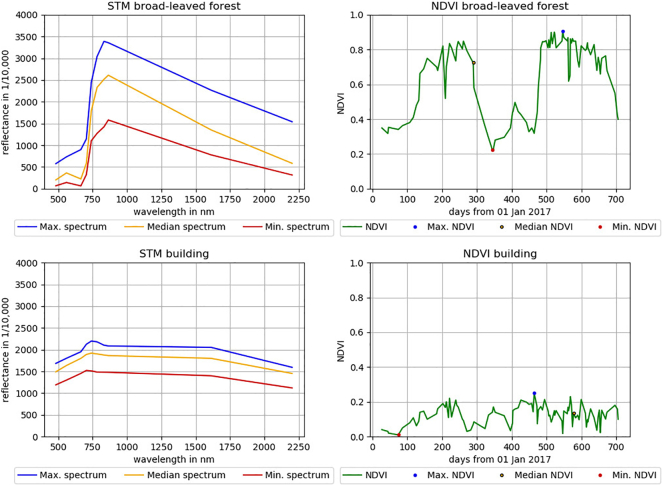
Left: Spectral-temporal metrics: Minimum, maximum and median spectra for a broad-leaved forest (top) and building pixel (bottom). Right: Maximum, minimum and median NDVI values for the same pixels over the two-year study period.

The two-year time series of Sentinel-2A/B images was used to extract 18 STM of reflectance and the NDVI. STM are pixel-based statistics of reflectance or derived indices of all clear-sky observations within a time period (Müller et al., 2015). Being a spectral index, we consider the temporal NDVI statistics as spectral-temporal metrics in the course of this study. We computed nine STM from the ten spectral bands and nine STM from the NDVI, including mean and spectral quantiles (minimum, 25th, 50th, 75th and maximum) as well as standard deviation (SD), range and inter-quartile range (IQR) (Fig. 2).

This yielded a total of 18 different *feature sets* (nine STM from reflectance with ten *features* (bands) each and nine STM from the NDVI with one feature each) which, in different combinations (*feature set combination*), served as an input to the regression model training.

(1) $$C_{n}^{m}=\frac{n!}{m!\left(n-m\right)!}$$

Eq. (1) describes all possible combinations C of drawing m feature sets from a set of n feature sets, while

(2) $$\sum_{m=1}^{n}C_{n}^{m}=\frac{n!}{m!\left(n-m\right)!}$$

Eq. (2) describes the total amount of possible combinations to be considered in our analysis. With n being 18, there will be 262,143 feature set combinations, each leading to a separate model per LC type. Due to processing time restrictions, and the likely degeneracy between different combinations of STM, we reduced the number of combinations by grouping feature sets with similar information into overall eight categories (Table 1).

**Table 1:** Groups of STM used for model training.

Group	Feature sets
Reflectance variation	SD, IQR, Range
Low reflectance	Minimum, 25th quantile
Medium reflectance	Mean, 50th quantile
High reflectance	75th quantile, Maximum
NDVI variation	SD, IQR, Range
Low NDVI	Minimum, 25th quantile
Medium NDVI	Mean, 50th quantile
High NDVI	75th quantile, Maximum

We drew feature set combinations including feature sets from one to eight randomly chosen groups. While there is one model using randomly chosen feature sets from all (eight) groups, there are eight models using a single feature set from each group. An additional example: There are 28 combinations of two groups (e.g. groups 1 and 2, 1 and 3, 1 and 4, …, 2 and 3, 2 and 4, …7 and 8). For each of the 28 group combinations, we randomly choose one feature set from each of the two groups. The total amount of possible feature set combinations from a set of eight groups is 255. Each of those combinations was individually used as an input to fraction modelling (Table 2). In order to cover more feature set combinations, the process was repeated five times, leading to a total of 1275 models with possible duplicates.

**Table 2:** Input data overview and selected feature set combinations with n = 8.

Feature sets per model (m)	Possible feature set combinations	Example feature set combination
1	8	One feature set from each group (e.g. Reflectance Min.)
2	28	Two random feature sets from one group each (e.g. Reflectance Mean & Range) using all 28 combinations of two groups
3	56	Reflectance Mean & Max. + NDVI Min.
4	70	Reflectance Mean & Range + NDVI Min. & 50th quantile
5	56	Reflectance Min. & IQR & 75th quantile + NDVI Max. & SD
6	28	Reflectance 25th quantile & Mean & Max. + NDVI Mean & Range & Max.
7	8	Reflectance 25th quantile & Mean & Max. & IQR + NDVI Min. & Mean & 75th quantile
8	1	Reflectance Min. & Mean & Range & 75th quantile + NDVI SD & Min. & Mean & Max.
	Total: 255	

### 3.2 Training data

We used a regression-based unmixing approach based on synthetically mixed training data from STM libraries. As we compared the performance of different models, each model needed a separate library with respect to its input feature set combination. The identification of reference sites for pure surfaces started in the greater Berlin-Brandenburg region including parts of adjacent federal states in north-east Germany (371 sites). A smaller number of sites was incrementally added in different regions all over Germany and Austria, since not all known LC types were found in the focus area, for example wetlands, surface coal mining, bare rock or slate roofs (85 sites). Seventy-eight permanent bare rock and soil locations were included as a background class for the synthetic mixing process. Reference sites were selected based on the visual interpretation from very high resolution (VHR) imagery in Google Earth and needed to feature invariant LC during the study period. The size of homogeneous reference objects was required to be at least 20 × 20 m, if possible, knowing that some LC is typically represented through smaller objects. Additional criteria for reference sites included: Built-up surfaces and infrastructure references must not exceed an NDVI of 0.3 over the study period. This threshold is widely used to broadly separate vegetated and non-vegetated areas (Liu and Juárez, 2010; Esau et al., 2016). In contrast, a vegetation reference must exceed an NDVI of 0.8 at least once within the study period. The NDVI saturates at closed canopy cover, i.e. pixels above that threshold most likely represent pure vegetation (Asrar et al., 1984). The same reference locations were used for all libraries that serve as input for the training of each LC-specific regression model. We chose a hierarchical library approach of three levels in order to account for intra-class variance and sampled reference locations for three Level-1, four Level-2 and nine Level-3 LC types (Table 3). For more complex LC types such as *built-up and infrastructure*, more spectra were sampled than for types with less surface variation, such as *woody vegetation*.

**Table 3:** Number of reference spectra by hierarchical class.

Level-1 class	Level-2 class	Level-3 class	Spectra
Built-up and infrastructure	Built-up	Clay tile Roof	51
		Dark Roof	42
		Bright Roof	51
	Infrastructure	Roads	37
		Railways	32
		Other open impervious spaces	45
Woody vegetation			90
Non-woody vegetation	Permanent		23
	Seasonal		85
Total			456

**Fig. 3:**
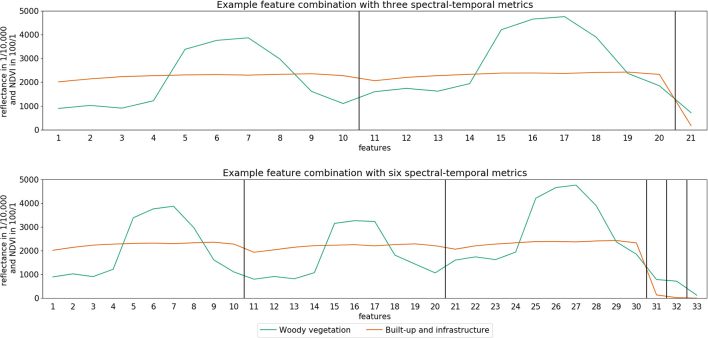
Representation of vegetation (green) and built-up and infrastructure (orange) STM for two selected feature set combinations. Top: Feature set combination with 21 features from mean reflectance (features 1–10), 75th percentile of reflectance (f. 11–20) and 25th percentile of the NDVI (f. 21) throughout the time series. Bottom: Feature set combination with 33 features from mean reflectance (f. 1–10), 25th (f. 11–20) and 75th percentile of reflectance (f. 21–30), the NDVI range (f. 31), the NDVI median (f. 32) and the 25th percentile of the NDVI (f. 33) throughout the time series (For interpretation of the references to color in this figure legend, the reader is referred to the web version of this article.)

One library was extracted per feature set combination, resulting in a number of 1275 libraries. The composition of a library varied according to the input feature sets and contains a different number of features per sample. Fig. 3 shows two examples of STM from different input libraries.

### 3.3 Regression-based unmixing and post-processing

Fraction mapping used target LC types of Level-1 of the classification hierarchy. We used a Kernel Ridge Regression (KRR) algorithm for fraction mapping. KRR is a machine learning regressor and applies linear regression models to high dimensional data that have been transformed based on non-linear kernels (Murphy, 2012). KRR is similar to Support Vector Regression (SVR) approaches that are a form of Support Vector Machines (SVM), but uses a different loss function. For our purpose, KRR is advantageous because of its higher computational performance compared to SVR (Verrelst et al., 2012). Kernel-based approaches have been shown to be useful in remote sensing applications (Mountrakis et al., 2011), particularly when dealing with high intra-class spectral variability (Rosentreter et al., 2017; Okujeni et al., 2017). The computational environment we used featured an Intel® Xeon® L7545 infrastructure with 24 cores/1.87 GHz and 640GB DDR3 RAM (1066 MHz). Our implementation the EnMAP-Box (EnMAP-Box Developers, 2019) API for Python.

**Fig. 4:**
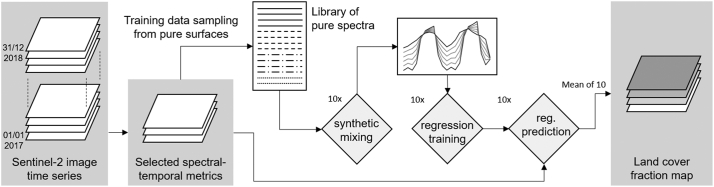
Basic workflow for KRR with synthetic mixing (workflow for one model). Libraries of pure spectra are drawn from image time series STM. Synthetic mixing uses those pure spectra to create artificial mixtures.

Spectral libraries provide collections of STM that represent pure LC types (hereafter: *pure STM*). Regression-based unmixing with synthetically mixed training data creates artificial mixtures of pure STM from different classes (inter-class and intra-class mixtures for inter-class and intra-class variability). These mixed STM together with the mixing ratio as label are then used as continuous input for regression training, i.e. independent and dependent variable, respectively (Fig. 4). We adapted a parameter setup that was previously used in local to regional settings (Okujeni et al., 2017; Okujeni et al., 2018; Schug et al., 2018). Twelve hundred artificial STM mixtures were created from each library of pure STM and used to train a model and predict LC fractions. Mixtures were produced with a randomized mixing approach that mixes pure STM at different binary and ternary ratios. To represent the variability of the sampling process, this procedure was repeated ten times. Prediction results of each prediction were averaged.

Results were masked using water, terrain slope and snow cover masks. The water mask was based on the Normalized Difference Water Index (NDWI, McFeeters, 1996) and was expanded by pixel reflectance criteria (suggested in Jones, 2019). Pixels with a value >0.1 in a two-year NDWI time series and with reflectance values lower than 0.035 in red, lower than 0.15 in NIR (Sentinel-2A/B band 7) and lower than 0.1 in SWIR band (band 11) were flagged as water. Slope was derived from the Shuttle Radar Topography Mission digital elevation model with a resolution of 1 arc-second (USGS, 2006). We masked pixels with a slope steeper than 35 degrees as built-up surfaces and infrastructure are not likely to occur there. We additionally masked all pixels where snow was detected in >50% of all observations. Those areas likely suffered from misleading intra-annual STM and are only found in high alpine regions.

### 3.4 Validation

**Fig. 5:**
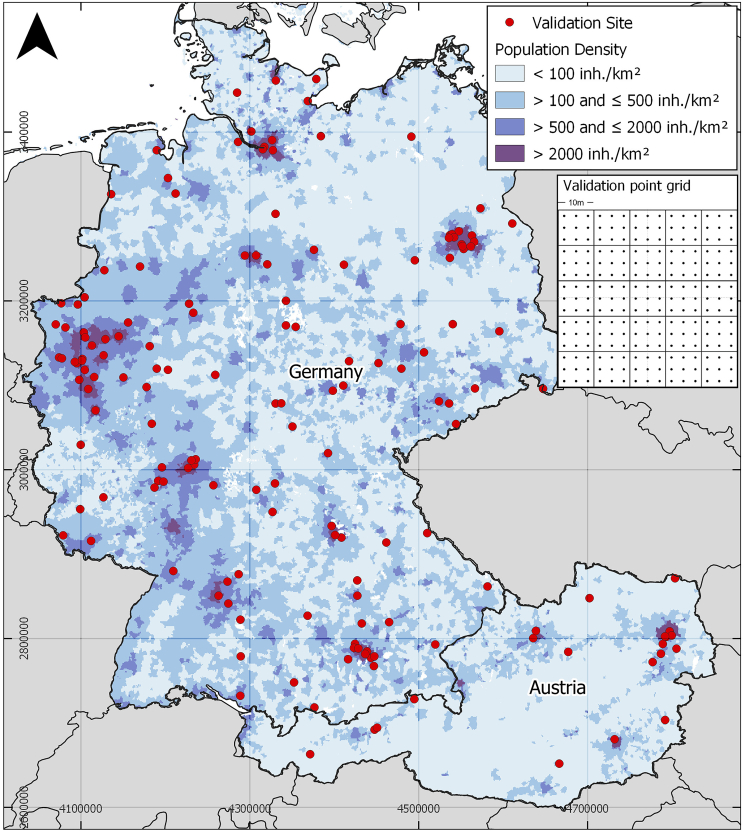
Study area with 160 validation sites and three population density classes used for hierarchical validation site stratification. Population density from (CIESIN, 2017). White surfaces: No data. Inset: Regular grid of validation points per site.

We conducted a systematic model performance assessment using independent validation sites across the study region. Those sites were sampled based on a two-step stratified random sampling approach (Olofsson et al., 2014) that aimed at placing them in both urban and rural contexts as well as on presumably built-up and non-built-up surfaces in order to cover all areas of interest of this study equally. First, population density from the Gridded Population of the World (GPW) layer by the Center for international Earth Science Information Network (CIESIN) in its fourth version (CIESIN, 2017) was used as a first 4-layer stratum (0–100, >100–500, > 500–2000 and >2000 inh./km^2^) in order to cover densely to less densely populated areas. The Copernicus Imperviousness Layer (Langanke et al., 2018) was used as a second stratum and proxy for built-up features. We defined four classes of imperviousness (0–0.25, >0.25–0.5, >0.5–0.75 and >0.75–1) in which we drew a respective number of 10 sample locations, which is a total of 40 sites in each population density stratum and a total of 160 validation sites in the study area. Fig. 5 illustrates the study area and the location of all validation sample areas.

Reference LC information was generated for the 160 sample locations and was derived from VHR imagery including imagery accessible in web map services (Google Earth, OpenStreetMap, MapBox). Each sample location displayed in Fig. 5 corresponds to the seed pixel of a reference grid (Fig. 5 inset). We collected pixel-wise fraction reference data with a systematic grid approach for 25 pixels of 10 × 10 m^2^ in each sampling area. Within each pixel, we label LC classes of 3 × 3 points. Using the Sentinel-2 raster grid, the labelled points were used to calculate LC fractions. The process of reference data collection encompasses 160 sites with 225 reference points each, yielding a total of 36,000 reference points. The performance of each model is evaluated comparing predicted LC fractions to reference fractions. Quality metrics for regression model evaluation included the model's root mean squared error (RMSE), mean absolute error (MAE), slope and intercept for measuring model accuracy and the coefficient of determination (R^2^) for measuring model precision. We validated results at a spatial resolution of 20 m and 50 m. the map was not validated at a 10 m spatial resolution, since map quality would be affected by the absolute geolocation error of Sentinel-2A/B of about 10–12 m (Gascon et al., 2017). Also, VHR imagery in web map services can have a similar geolocation error (Goudarzi and Landry, 2017). We consider models to be useful for mapping large area LC fractions if quality measures are comparable to those of other studies using a similar methodology on a local level or at coarser resolutions (e.g. Walton, 2008, Okujeni et al., 2018 or Priem et al., 2019, achieving an MAE range of 0.08 to 0.13 at resolutions of 30 m - 60 m)

### 3.5 Selection of a feature set combination for nation-wide mapping

Finally, a single feature set combination was chosen to map LC fractions for the whole study area. This model selection was largely driven by comparative model performance. We aimed at selecting an STM feature set combination that is useful for creating synthetic training data and that produces balanced and good results across classes. A weighted quality score was calculated based on the following criteria:

1) Models with a good performance across all classes must have a high score. Models particularly under-performing in at least one class must be penalized.
2) A good performance implies comparatively good values with regard to RMSE, slope and R^2^.
3) The score standardizes the different scales of quality metrics.

Quality scores were summed for each measure and each class. Our weighted score was based on the logarithmic transformation function

(3) $$f\left(x\right)=\frac{1}{4}\;\log_{10}\left(x+0.001\right)+1$$

with x as the normalized RMSE, 1/R^2^ and |(1 - Slope)| for each model respectively. We added 0.001 for the function to be defined for x = 0.

## 4 Results

### 4.1 Land cover fractions model quality

Model quality assessment included 1275 models from which 162 were duplicates due to the random selection of input feature set combinations. Computation time of one KRR model ensemble training with 30 features was around 18 min on a single core.

**Fig. 6:**
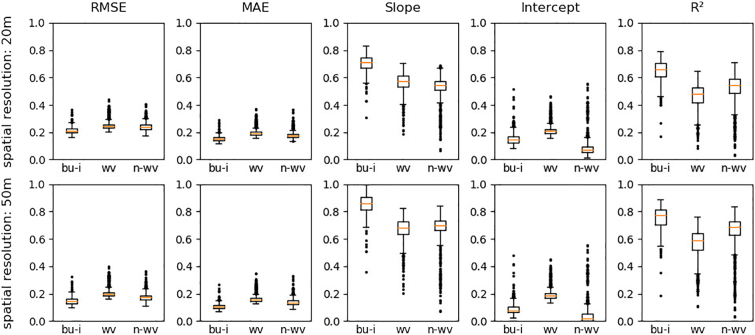
Quality measures (RMSE, MAE, Slope, Intercept, R^2^) for all studied models (*n* = 1113), all classes, spatial resolution of 20 m and 50 m. Built-up and infrastructure = bu-i, Woody vegetation = wv, Non-woody Vegetation = n-wv. Outliers: lower than 3rd quartile + 1.5 ∗ inter-quartile range and greater than 1st quartile − 1.5 ∗ inter-quartile range (Hunter et al., 2017).

Validated at a resolution of 20 m (Fig. 6), the distributions of the selected quality metrics excluding outliers showed that for *built-up and infrastructure*, *woody vegetation* and *non-woody vegetation*, the MAE values of all models were respectively close to each other, ranging from 0.11 to 0.20 (*built-up and infrastructure*), 0.15 to 0.23 (*woody vegetation*) and 0.13 to 0.22 (*non-woody vegetation*). All classes had outliers with higher MAE. RMSE showed a similar distribution with higher values compared to MAE. Slope and intercept of the regression models showed a similar compact distribution with larger between-class differences of *built-up and infrastructure* (median slope/intercept of 0.73/0.14), *woody* (median slope/intercept of 0.57/0.20) and *non-woody vegetation* (median slope/intercept of 0.55/0.07) and with few outliers featuring higher and a greater number of outliers with lower quality. R^2^ ranged from 0.46 to 0.85 for *built-up and infrastructure*, from 0.26 to 0.71 for *woody vegetation* and from 0.33 to 0.79 for *non-woody vegetation* models. Validated at an aggregated resolution of 50 m, the patterns of the examined quality metrics were similar with a higher overall quality.

**Fig. 7:**
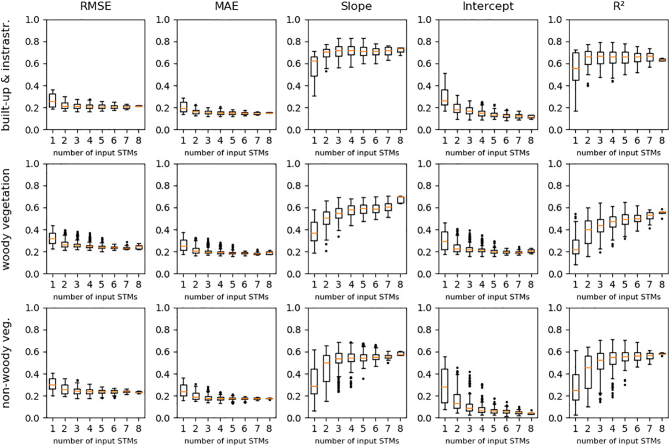
RMSE, MAE, Slope, intercept and R^2^ for all models and classes with regard to the number of STM inputs. Spatial resolution: 20 m.

Fig. 7 illustrates that models with a single STM feature set input provided lower quality than models with two or more input feature sets. Two to four feature sets for regression training and prediction was sufficient to reach comparatively good results with regard to RMSE, MAE, slope, intercept and R^2^. A higher number of input feature sets in most cases did not lead to an additional gain in quality. None of the potential input STM had a negative impact on model quality by its presence among the input feature sets.

### 4.2 Selection of a feature set combination for nation-wide mapping

Six hundred and sixty models were based on four or less feature sets. A ranking of model qualities shows that there were no models that performed consistently well across all classes and quality metrics, and that for individual classes and quality measures there were also a large number of models that performed similarly well (Fig. 6).

**Fig. 8:**
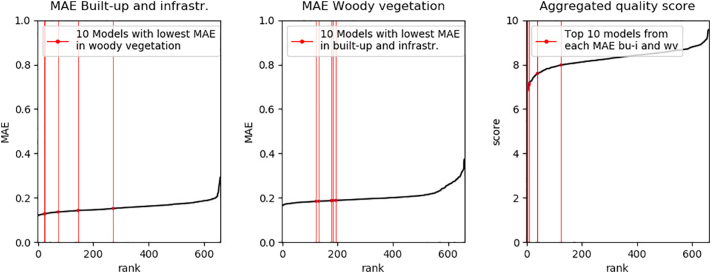
Left: Models with four or less input variables sorted by MAE. Class Built-up and infrastructure. Center: Models with four or less input variables sorted by MAE. Class Woody vegetation. Right: Aggregated quality score of three classes and three quality measures (MAE, Slope, R^2^). Spatial resolution: 20 m.

We found that the difference in quality among the best performing models was rather low when considering single LC types or quality metrics and that models that performed best for one LC type or in one metric could be ranked lower in a different class or metric (Fig. 8 left and center). The differences in model quality were more distinct in the weighted ranking (Fig. 8 right). While, in the example, MAE differences between the best models are rather low, differences in the models with best aggregated quality score were clearly higher.

Models from two feature set combinations were within the top 5% of the aggregated quality score and also within the top 10% of all class-wise quality scores (Table 4).

**Table 4:** Top 15 models based on the overall quality score. Models in **bold** were within the Top 5% of the quality score and also within the Top 10% of all class-wise quality scores. bu-i = built-up & infrastructure, wv = woody vegetation, n-wv = non-woody vegetation. Ref. = Reflectance, Q. = Quantile.

Feature set combination	Score bu-i	Score wv	Score n-wv	Score ∑
IQR Ref./25th Q. Ref./NDVI IQR/NDVI Max	2.31	2.79	1.16	6.27
50th Q. Ref./NDVI IQR/25th Q. NDVI/75th Q. NDVI	1.09	2.78	2.63	6.52
50th Q. Ref./NDVI IQR/50th Q. NDVI/75th Q. NDVI	1.44	2.76	2.60	6.82
Mean Ref./STDEV NDVI/Mean NDVI/Max Ref.	**1.54**	**2.71**	**2.58**	**6.84**
50th Q. Ref./NDVI IQR/75th Q. NDVI	1.48	2.79	2.65	6.92
Mean Ref./75th Q. NDVI	2.14	1.92	2.89	6.96
50th Q. Ref./50th Q. NDVI/75th Q. NDVI	1.50	2.82	2.72	7.05
Min Ref./75th Q. Ref./75th Q. NDVI	2.57	1.88	2.60	7.06
50th Q. Ref./75th Q. Ref./75th Q. NDVI	1.98	2.35	2.73	7.07
50th Q. Ref./Mean NDVI/25th Q. NDVI/75th Q. NDVI	1.67	2.77	2.63	7.07
50th Q. Ref./75th Q. Ref./NDVI Range/75th Q. NDVI	1.96	2.39	2.77	7.13
50th Q. Ref./25th Q. Ref./75th Q. Ref./NDVI MAX	**2.18**	**2.51**	**2.44**	**7.14**
STDEV Ref./50th Q. Ref./Mean NDVI/NDVI Max	1.54	2.82	2.84	7.21
50th Q. Ref./75th Q. Ref./Mean NDVI/75th Q. NDVI	1.77	2.67	2.77	7.22
50th Q. Ref./Mean NDVI/75th Q. NDVI	1.68	2.82	2.72	7.23

### 4.3 Nation-wide land cover fraction mapping

**Fig. 9:**
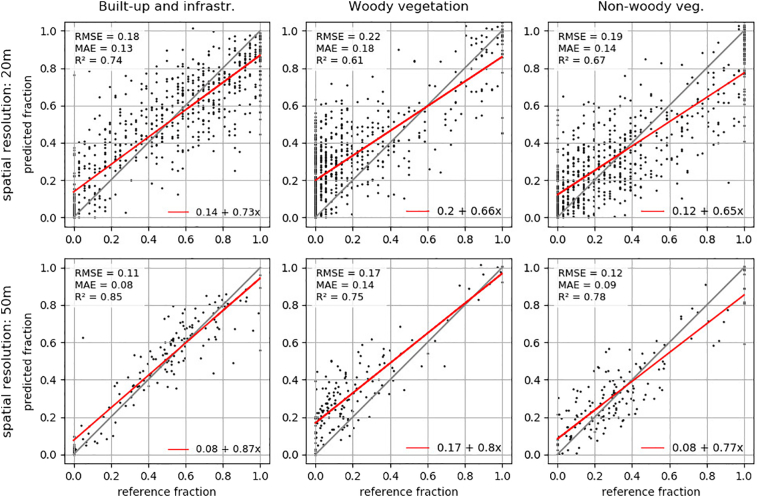
Scatterplots of regression predictions of the selected model (with 25th and 75th percentile of reflectance, median reflectance and maximum NDVI) for two spatial resolutions (20 m, 50 m) and all classes.

For nation-wide mapping, we chose a model based on median reflectance, 25th percentile of reflectance, 75th percentile of reflectance and maximum NDVI. With regard to the metrics groups established before model training, this model covered information from low, medium and high spectral reflectance. The 25th and 75th percentile of reflectance also implicitly included spectral variation. Among the suggested NDVI metrics, higher NDVI metrics were the ones that occur most often in the best performing models. Fig. 9 illustrates the regression quality of this model for all LC types. Computation time for the prediction of LC fractions for a 30 × 30 km tile was about 430 min using KRR (on a single core).

**Fig. 10:**
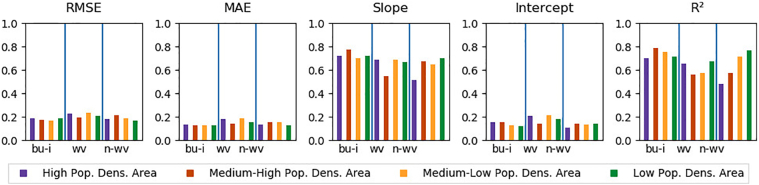
RMSE, MAE, Slope, Intercept and R^2^ for all models and classes at a spatial resolutions of 20 m stratified by population density. bu-i = Built-up and infrastructure, wv = Woody vegetation, n-wv = Non-woody vegetation.

Validating the selected model stratified by the four population density classes used for validation site selection showed that quality metrics varied regionally. Whereas RMSE, MAE and intercept were relatively stable across all classes, slope had lower values for woody and non-woody vegetation in high and medium-high density areas. R^2^ had better values for woody vegetation in a high and low density areas and weaker values for non-woody vegetation in high population density areas (Fig. 10).

**Fig. 11:**
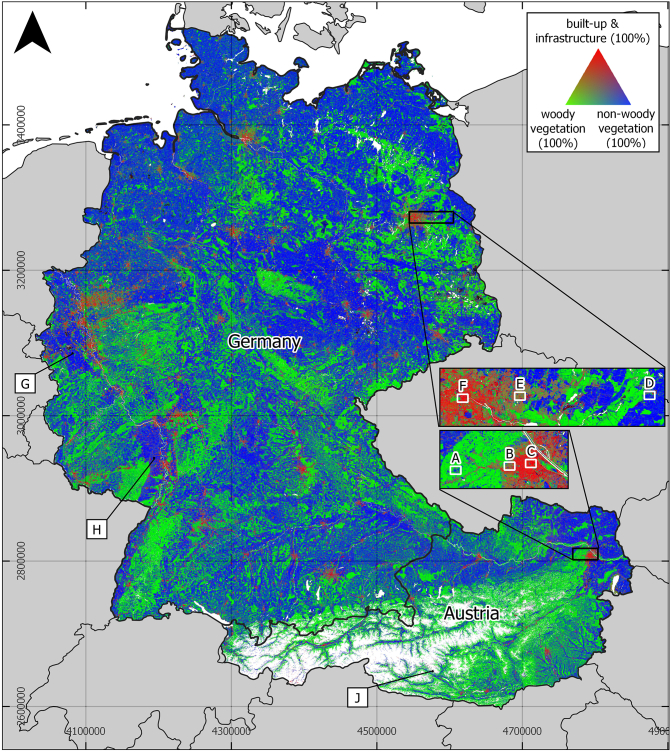
Land cover fractions of built-up and infrastructure (red), woody vegetation (green) and non-woody vegetation (blue) in Germany and Austria. White pixels: Masked pixels due to the occurrence of water, permanent snow or steep slope. Focus areas A to J will be described in Fig. 12, Fig. 13. (For interpretation of the references to color in this figure legend, the reader is referred to the web version of this article.)

Fig. 11 shows a nation-wide LC fraction map of *built-up surfaces and infrastructure*, *woody vegetation* and *non-woody vegetation* in Germany and Austria. The overall share of built-up surfaces and infrastructure is 9.6% in Germany and 6.7% in Austria, while woody-vegetation covers 40.6% / 40.7% and non-woody vegetation covers 54.8% / 31.9% of the total surface area. Remaining fractions are covered by permanent soil or have been masked. Even though not congruent in their definition, the resulting LC shares of *built-up surfaces and infrastructure* approach those shares found for *artificial areas* in the CORINE land cover product of 2018 (9.4%/5.9%). Shares of *woody vegetation* do not correspond to the shares given for *forest* in CORINE (31.6% / 54.0%). Shares of *non-woody vegetation* are, again, comparable to the share of *agricultural land* (57.3% / 31.9%) in CORINE.

**Fig. 12:**
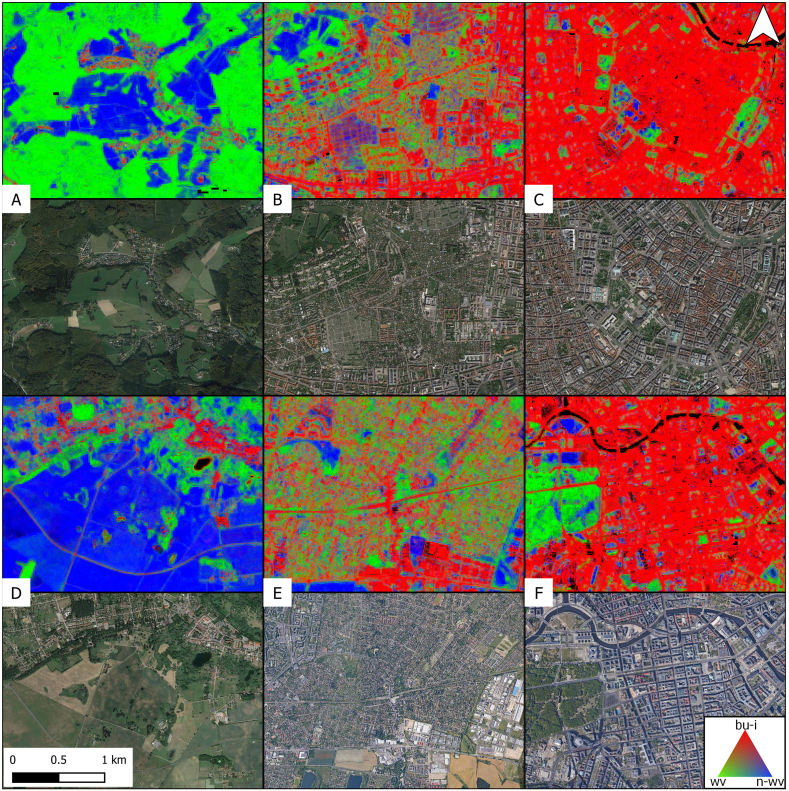
Land cover fractions of built-up and infrastructure, woody vegetation and non-woody vegetation (top) in six selected areas (c.f. Fig. 11) along the rural-urban gradient. Bottom: True color very high resolution image from Google Earth. Bu-I = built-up and infrastructure, wv = woody vegetation, n-wv = non-woody vegetation. Black: Masked areas.

In the Austrian and German Alps, larger areas were masked because of permanent snow cover and steep slopes. Apart from that, only a small fraction of the study area was masked because of surface water. LC composition varies according to the respective region and large natural features and geographic regions can be distinguished even at a broad scale. Fig. 12 is an insight into six smaller areas representing a rural-urban gradient in two parts of the study area. We observe that the urban core areas of Vienna and Berlin (Fig. 12C and F) are largely characterized by built-up surfaces and infrastructure. Urban parks and forests stand out by their increased fraction of woody and non-woody vegetation. Fig. 12B and E are representations of populated areas at the urban fringe of Vienna and Berlin, where an increased number of mixed pixels and a higher heterogeneity of LC patterns is found. If not surrounded by buildings, roads and railways stand out through their linear character. In rural areas (Fig. 12A and D), large built-up surfaces are rather rare. Instead, smaller settlement features become visible as lower fraction values. In rare cases, very small water patches were not detected by the water mask and were mostly given a high fraction of built-up surfaces and infrastructure (Fig. 12D).

**Fig. 13:**
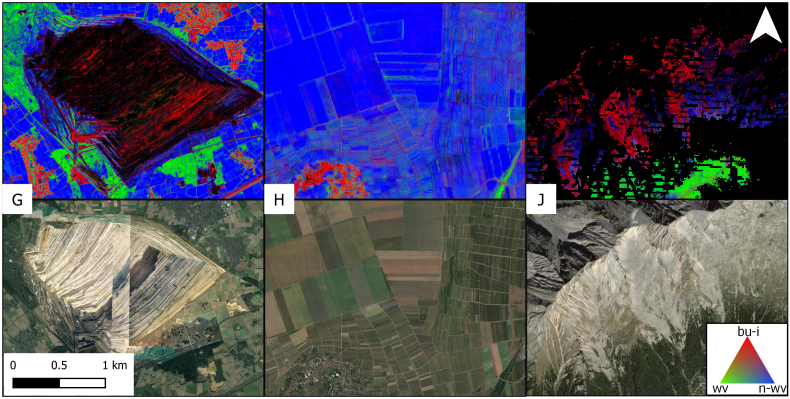
Land cover fractions of built-up and infrastructure, woody vegetation and non-woody vegetation (top) in areas (c.f. Fig. 11) shaped by open coal mining (G), vineyards (H) and mountainous bare rock (J). Black: Masked areas. Black stripes in J are masked terrain slopes above 35 degrees. Bottom: True color VHR image chips from Google Earth.

Apart from settlement areas, fraction mapping provided good results in other landscapes including forests, agricultural areas or grasslands, too. However, some areas featuring relatively rare LU types showed incorrect LC abundance. Fig. 13G, H and J are examples for LC fractions in an open coal pit (G), in vineyards (H) and for mountainous bare rock surfaces (J). Here, fractions of built-up and infrastructure surfaces reached up to 0.4 (H) and 1 (G, J) respectively. Parts of the areas in G and J were masked.

## 5 Discussion

### 5.1 Spectral-temporal metrics for land cover fraction mapping

Regression-based unmixing using synthetic training data for LC fraction mapping has mainly been applied on a local and regional level. In this study, we successfully extended the approach to a national scale. We showed that temporal metrics of Sentinel-2 A/B time series reflectance and derived indices can be successfully used to generate synthetic training data for regression modelling. The approach combined the benefits of STM, i.e. their temporal and spatial spectral robustness with regard to phenology and noisy or missing data, with the benefits of synthetic training data, i.e. the need of only pure reference spectra and comparatively few training data. We used synthetically mixed training data with STM in contrast to previous studies using spectra from a single observation (Okujeni et al., 2017), bi-seasonal imagery (Schug et al., 2018) or best-pixel composites (Suess et al., 2018). The quality of the best performing models was consistent with the model quality found in previous studies with synthetic training data and other regression modelling, acknowledging that model input data, class complexity and spatial resolutions are different (MAE 0.12 for mapping imperviousness in Walton (2008) using SVR at 30 m resolution, average MAE of 0.08 to 0.13 for local models mapping V-I-S in Okujeni et al. (2018), using SVR at 30 m resolution, MAE of 0.09 to 0.1 in Priem et al. (2019) using SVR validated at 60 m resolution). However, we achieved a similar MAE while applying our analysis in a nation-wide wall-to-wall setup.

Regression results were validated based on common quality metrics for regression analysis and a comparative approach showed that there is a set of models based on different STM feature set combinations that perform similarly well. Even in a scored ranking, the differences in overall model quality were low, indicating the high robustness of STM as regression input for large-area studies. It appeared that representing spectral variability throughout the study period based on STM allows for characterizing urban-rural and environmental gradients well between the Baltic Sea in the North and the Alps in the South. Most of the best performing models also included NDVI-based information. Using a maximum NDVI facilitated the distinction of seasonally vegetated surfaces in the multispectral-temporal feature space.

We found that three to four STM feature sets for regression training were sufficient to reach the best model quality. A greater number of feature sets did not improve the overall model quality. That applies for nation-wide mapping and is in line with results from rather local studies. For example, Schneider (2012) found that a relevant data reduction, i.e. only using the top 50% features determined with Random Forest feature importance, did not lead to a decrease in quality compared to using all features in urban LC mapping with SVR. While not all theoretically possible STM feature set combinations could be tested, the available spectral-temporal feature space was well covered, proven by the fact that various models reached good results.

Considering spatial resolution, we confirmed that our method allows mapping LC fractions with a resolution as fine as 10 m. Potential geolocation errors of Sentinel-2A/B and Google Earth imagery, however, can add up to >12 m and lead to noise and associated uncertainties that prohibit model validation at 10 m resolution. Sentinel's geolocation error also applies to the resulting maps; however, such effects are likely to decrease once the improved Sentinel-2 geo-registration based on the Global Reference Image (GRI) is available (Gascon, 2019). In addition, our sampling design only permitted reference LC fractions in steps of 11.1% at a resolution of 10 m, as there were nine interactively collected reference labels within one pixel of 10 × 10 m^2^. This prompted us to use a more robust validation procedure at 20 m. Also, the general tendency to reduce model errors by prediction averaging, i.e. smoothing, contributed to the increased quality at coarser resolutions. The stratification strategy for collecting validation data was a two-step approach based on population density and imperviousness from GPW and the Copernicus Imperviousness Layer. Knowing that the GPW distributes population equally within the administrative spatial units (CIESIN, 2017), a population dataset using a more dynamic and possibly more accurate distribution could enhance the validation strategy in place. In addition, while we observed a good distribution of reference LC fractions for built-up surfaces and infrastructure, this distribution seemed to be left-skewed for the two vegetation classes. This was likely due to our sampling strategy relying on two datasets that both target settlements and impervious surfaces in particular. Further studies might benefit from a sampling approach that covers the whole range of woody and non-woody vegetation fractions more equally.

Spatially stratified quality assessment by population density showed that the map quality of built-up and infrastructure areas was fairly stable across the three LC types. Larger differences could be observed in woody and non-woody vegetation. Non-woody vegetation tended to show lower model quality (Slope, R^2^) in high and medium-high density areas. A reason for that might be a larger impact of illumination variance and shadow on low non-woody vegetation in urban areas, making the surface resemble to darker woody vegetation. Also, patch sizes of vegetation are smaller in populated areas (mostly parks and open grass patches), the variation of vegetation fractions and, subsequently, the impact of image geolocation errors are higher. From a methodological perspective, this might also be caused by the built-up centric sampling design underrepresenting rare woody vegetation in densely populated regions.

With regard to our first research objective, aiming at the combined use of STM with synthetic training data for LC fraction mapping, we showed that STM are a highly suitable input for creating synthetically mixed training data. Using synthetic mixtures of STM, where none of the feature sets is an actually measured spectrum in an image, produced results comparable to local and regional studies using training data from an actual image and allows to map land cover with single models consistently over large areas.

Considering our second research objective, aiming to evaluate the performance of STM combinations, we state that multiple STM feature set combinations produced similarly good results. Various STM feature set combinations are able to accurately quantify LC.

### 5.2 Nation-wide land cover fraction mapping

While selected single images or image composites might perform equally in LC fraction mapping, the use of STM facilitates large area mapping. Using STM, no particular data selection process is necessary. STM contain information on vegetation phenology, which offers great benefits when mapping surfaces with seasonal characteristics. This is specifically advantageous to mitigate differences in phenology along north-south trajectories or due to regional climatic conditions or topography. Each of these factors applies to our region of interest with its strong latitudinal and elevation gradients. Using STM reduces both the impact of data noise and data gaps. We showed that many STM feature set combinations perform well as an input to regression modelling. Using multi-spectral data, the feature space of a synthetically mixed regional library of pure STM is sufficient for mapping a large area with similar LC characteristics. Water surfaces were not included in the validation process, leading to errors when the water mask was not accurate. High fractions of built-up surface where small water bodies were present could be explained by the spectral ambiguity between water and surfaces with dark shadow pixels in urban areas that were selected as pure references and part of the synthetic mixing procedure.

Our broad-scale mapping on a nation-wide level (covering an area of 440.000km^2^ with 86 Sentinel-2 MGRS tiles) showed that model prediction challenges exist in areas with rare LC and LU types, such as mining sites, beaches, vineyards or mountainous bare rock surfaces. The error in all these cases is related to the permanent occurrence of soil and rock surfaces and their spectral-temporal similarity to built-up surfaces. Previous large-area LC maps with a different mapping focus (Griffiths et al., 2019; Pflugmacher et al., 2019) have documented similar issues. The confusion of soil with built-up surfaces and infrastructure might imply that the data feature space is actually representing non-vegetated area instead of built-up features and infrastructure. That does not seriously affect the overall map quality in temperate regions, because of the rare occurrence of permanent open soil. It does, however, underline the importance of STM as they improve our ability to reliably exclude temporary soil surfaces, such as in agriculture or deciduous forests. However, this potential confusion might become a severe challenge when using the approach in regions with higher proportions of bare soil and rock surfaces, for example in the Mediterranean or other semi-arid to arid regions. Further research is therefore required on the regional transferability of the approach. Moreover, the effect of additional data (e.g. Sentinel-1) on the procedure of synthetically mixing pure STM has not been explored here. This is highly relevant in order to tackle questions of urbanization in areas where optical data can be too sparse to allow for robust STM, such as in the tropics or mountainous regions. A discussion of target LC types is also relevant with regard to comparing the resulting maps with existing products: While many approaches focus on better mapping and separating built-up from all other LC (e.g. GHSL), we also wish to capture other surfaces that are inherent components of settlements (e.g. roads or vegetation). In contrast to studies with an urban focus, we represent the complete gradient from urban to rural settlements and offer a map that can help drawing a more complete and more granular image of settlement structure for urban ecological research. Current model computing time does not suggest efficient scalability to continental or global applications. Mapping our study area on the system described above took about 7.5 days. However, as the application scales well with used computing units, this is easily reducible on local infrastructures already. An implementation on cloud computing platforms or in a different programming environment (e.g. C instead of Python) could increase performance significantly and would allow for a re-evaluation of scalability.

The topic of spectral library creation and usage is of high interest to the research community (Somers et al., 2011; Deng, 2016; Degerickx et al., 2017). Here, we iteratively and manually developed a library of STM and achieved our results with a library representing surfaces of a sub-region of the study area. It was beneficial that the study area is located within similar ecoregions and that data was consistent and similarly available. However, the question of how to ensure a representative library over larger areas through (semi-)automated approaches remains open and requires further research. We assume that due to regional surface specificities, ecoregions could be a better spatial unit for model stratification than national borders. In order to find large area training data, a strategy for assuring training data representativity for all LC types including rare surfaces is required. We see the need for a methodology that identifies representative training data across a large study area, quantifies their representativity and delineates sub-regions where this training data leads to good quality LC fraction estimates. That is particularly important for potentially continental or global applications, where a manual selection of models is not feasible.

The benefit of fraction mapping is most obvious in areas where sub-pixel objects are common and spectral heterogeneity is high. In our study area, different object sizes and spectral characteristics are represented in highly heterogeneous settlement structures. Fractions provide information beyond discrete LC classifications and particularly support the identification and characterization of sub-pixel features. With regard to our third research objective, aiming at the contribution of LC fraction mapping to understanding settlement type and structures in both urban areas, rural areas and their transition, we state that accurate LC fraction mapping can provide a more continuous perspective on settlement gradients. Sub-pixel distribution of built-up areas and infrastructure will help to rather study density instead of presence. Fractions of vegetation types contribute to an understanding of settlement surface composition, settlement greenness, accessibility of green areas, and the kind of vegetation might indicate its function.

## 6 Conclusion

This study uses regression-based modelling of nation-wide LC fractions with synthetically mixed training data in a temperate environment. We showed that Sentinel-2A/B spectral-temporal metrics allow us to extend the concept of synthetically mixed training data with a temporal component that is robust with regard to phenology and data gaps. The combined benefits of spectral-temporal metrics and synthetic training data allow to quantify multi-class fractions at 10 m resolution with a library mainly based on regional reference data. The model robustness renders our approach usable for national-scale research. At the same time, the approach is less susceptible to varying feature quality and data availability. Our setup is equally suitable for mapping urban core areas and rural settlements and is likely to yield similar results when applied to nations with similar environmental and architectural settings. It allows to identify distinct characteristics of settlement features, such as surface composition, built-up density or green space distribution. Understanding the quantitative land cover composition of settlements helps to monitor densification and growth processes in an urban-rural gradient. We emphasize the relevance of nation-wide mapping since it makes remote sensing data fully compatible to many existing socio-economic data sets and sources. Our results offer the potential to enhance existing global products by, for example, providing fine-resolution built-up surface density. We assume the maps to be useful for a characterization of settlements, enhancing frameworks of settlement categorization, or to support urban ecology studies in the light of settlement expansion or climate change adaptation.

## CRediT authorship contribution statement

**Franz Schug:**Conceptualization, Methodology, Software, Validation, Writing - original draft, Visualization, Investigation.**David Frantz:**Conceptualization, Writing - review & editing.**Akpona Okujeni:Methodology,**Software, Writing - review & editing.**Sebastian van der Linden:**Conceptualization, Writing - review & editing.**Patrick Hostert:**Writing - review & editing.

## Declaration of competing interest

The authors declare that they have no known competing financial interests or personal relationships that could have appeared to influence the work reported in this paper.

## References

[bb0005] Asner G.P., Knapp D.E., Cooper A.N., Bustamante M.M.C., Olander L.P. (2005). Ecosystem structure throughout the Brazilian Amazon from Landsat observations and automated spectral unmixing. Earth Interact..

[bb0010] Asrar G., Fuchs M., Kanemasu E.T., Hatfield J.L. (1984). Estimating absorbed photosynthetic radiation and leaf area index from spectral reflectance in Wheat1. Agron. J..

[bb0015] Baiocchi G., Creutzig F., Minx J., Pichler P.-P. (2015). A spatial typology of human settlements and their CO2 emissions in England. Glob. Environ. Chang..

[bb0020] Borak J.S., Lambin E.F., Strahler A.H. (2000). The use of temporal metrics for land cover change detection at coarse spatial scales. Int. J. Remote Sens..

[bb0025] Brown de Colstoun E.C., Huang C., Wang P., Tilton J.C., Tan B., Phillips J. (2017). Global Man-Made Impervious Surface (GMIS) Dataset from Landsat.

[bb0030] CIESIN (2017). Gridded population of the world, version 4 (GPWv4). Population Density Adjusted to Match 2015 Revision of UN WPP Country Totals, Revision 10.

[bb0035] Corbane C., Pesaresi M., Kemper T., Politis P., Florczyk A.J., Syrris V. (2019). Automated global delineation of human settlements from 40 years of Landsat satellite data archives. Big Earth Data.

[bb0040] DeFries R.S., Townshend J.R.G., Hansen M.C. (1999). Continuous fields of vegetation characteristics at the global scale at 1-km resolution. J. Geophys. Res..

[bb0045] Degerickx J., Okujeni A., Iordache M.-D., Hermy M., van der Linden S., Somers B. (2017). A novel spectral library pruning technique for spectral unmixing of urban land cover. Remote Sens..

[bb0050] Deng C. (2016). Automated construction of multiple regional libraries for neighborhoodwise local multiple endmember unmixing. IEEE J. Sel. Top. Appl. Earth Observations Remote Sensing.

[bb0055] EEA (2007). CLC2006 Technical Guidelines.

[bb0060] EEA (Ed.) (2019). CORINE land cover 2018. https://land.copernicus.eu/pan-european/corine-land-cover/clc2018.

[bb0065] EnMAP-Box Developers (2019). EnMAP-box. A QGIS plugin to process and visualize hyperspectral remote sensing data. Version 3. https://enmap-box.readthedocs.io/en/latest/.

[bb0070] ESA Earth Online (2017). First sentinel-2B images delivered by laser. http://tiny.cc/98p8gz.

[bb0075] ESA Sentinel Online (Ed.) (2018). Revisit and Coverage. https://earth.esa.int/web/sentinel/user-guides/sentinel-2-msi/revisit-coverage.

[bb0080] Esau I., Miles V., Davy R., Miles M.W., Kurchatova A. (2016). Trends in normalized difference vegetation index (NDVI) associated with urban development in northern West Siberia. Atmos. Chem. Phys..

[bb0085] Esch T., Marconcini M., Felbier A., Roth A., Heldens W., Huber M. (2013). Urban footprint processor—Fully automated processing chain generating settlement masks from global data of the TanDEM-X mission. IEEE Geosci. Remote Sensing Lett..

[bb0090] Foley J.A., Defries R., Asner G.P., Barford C., Gordon Bonan, Carpenter Stephen R. (2005). Global consequences of land use. Science (New York, N.Y.).

[bb0095] Frantz D. (2019). FORCE—Landsat + Sentinel-2 analysis ready data and beyond. Remote Sens..

[bb0100] Frantz D., Roder A., Stellmes M., Hill J. (2016). An operational radiometric Landsat preprocessing framework for large-area time series applications. IEEE Trans. Geosci. Remote Sensing.

[bb0105] Frantz D., Stellmes M., Roder A., Udelhoven T., Mader S., Hill J. (2016). Improving the spatial resolution of land surface phenology by fusing medium- and coarse-resolution inputs. IEEE Trans. Geosci. Remote Sensing.

[bb0110] Frantz D., Haß E., Uhl A., Stoffels J., Hill J. (2018). Improvement of the Fmask algorithm for Sentinel-2 images. Separating clouds from bright surfaces based on parallax effects. Remote Sens. Environ..

[bb0115] Gascon F. (2019). Sentinel-2 Mission Status.

[bb0120] Gascon F., Bouzinac C., Thépaut O., Jung M., Francesconi B., Louis J. (2017). Copernicus sentinel-2A calibration and products validation status. Remote Sens..

[bb0125] Gong P., Liu H., Zhang M., Li C., Wang J., Huang H. (2019). Stable classification with limited sample: transferring a 30-m resolution sample set collected in 2015 to mapping 10-m resolution global land cover in 2017. Science Bulletin.

[bb0130] Goudarzi M.A., Landry R. (2017). Assessing horizontal positional accuracy of google earth imagery in the city of Montreal, Canada. Geodesy and cartography.

[bb0135] Griffiths P., Nendel C., Hostert P. (2019). Intra-annual reflectance composites from Sentinel-2 and Landsat for national-scale crop and land cover mapping. Remote Sens. Environ..

[bb0140] Hansen M.C., DeFries R.S., Townshend J.R.G., Carroll M., Dimiceli C., Sohlberg R.A. (2003). Global percent tree cover at a spatial resolution of 500 meters. First results of the MODIS vegetation continuous fields algorithm. Earth Interact..

[bb0145] Herold M., Roberts D.A., Gardner M.E., Dennison P.E. (2004). Spectrometry for urban area remote sensing—Development and analysis of a spectral library from 350 to 2400 nm. Remote Sens. Environ..

[bb0150] Homer C., Huang C., Yang L., Wylie B., Coan M. (2004). Development of a 2001 National Land-Cover Database for the United States. Photogramm Eng Remote Sensing.

[bb0155] Hunter J., Dale D., Firing E. (2017). matplotlib.pyplot Documentation. https://matplotlib.org/api/pyplot_api.html#matplotlib.pyplot.boxplot.

[bb0160] Jones J. (2019). Improved automated detection of subpixel-scale inundation—revised dynamic surface water extent (DSWE) partial surface water tests. Remote Sens..

[bb0165] Kaspersen P., Fensholt R., Drews M. (2015). Using Landsat vegetation indices to estimate impervious surface fractions for European cities. Remote Sens..

[bb0170] Klotz M., Kemper T., Geiß C., Esch T., Taubenböck H. (2016). How good is the map? A multi-scale cross-comparison framework for global settlement layers: evidence from Central Europe. Remote Sens. Environ..

[bb0175] Lambin E.F., Geist H. (2006). Land-Use and Land-Cover Change. Local Processes and Global Impacts.

[bb0180] Langanke T., Steidl M., Schleicher C., Sannier C. (2018). Copernicus Land Monitoring Service - High Resolution Layer Imperviousness. Product Specifications Document.

[bb0185] Li J., Roy D. (2017). A global analysis of sentinel-2A, sentinel-2B and Landsat-8 data revisit intervals and implications for terrestrial monitoring. Remote Sens..

[bb0190] Liu W.T., Juárez R.I. Negrón (2010). ENSO drought onset prediction in Northeast Brazil using NDVI. Int. J. Remote Sens..

[bb0195] MacLachlan A., Roberts G., Biggs E., Boruff B. (2017). Subpixel land-cover classification for improved urban area estimates using Landsat. Int. J. Remote Sens..

[bb0200] McFeeters S.K. (1996). The use of the Normalized Difference Water Index (NDWI) in the delineation of open water features. Int. J. Remote Sens..

[bb0205] Melchiorri M., Florczyk A.J., Freire S., Ehrlich D., Schiavina M., Pesaresi M., Kemper T. (2018). Megacities Spatiotemporal Dynamics Monitored with the Global Human Settlement Layer.

[bb0210] Mountrakis G., Im J., Ogole C. (2011). Support vector machines in remote sensing. A review. ISPRS J. Photogramm. Remote Sens..

[bb0215] Müller H., Rufin P., Griffiths P., Barros Siqueira A.J., Hostert P. (2015). Mining dense Landsat time series for separating cropland and pasture in a heterogeneous Brazilian savanna landscape. Remote Sens. Environ..

[bb0220] Murphy K.P. (2012). Machine Learning. A Probabilistic Perspective.

[bb0225] Okujeni A., van der Linden S., Tits L., Somers B., Hostert P. (2013). Support vector regression and synthetically mixed training data for quantifying urban land cover. Remote Sens. Environ..

[bb0230] Okujeni A., van der Linden S., Hostert P. (2015). Extending the vegetation–impervious–soil model using simulated EnMAP data and machine learning. Remote Sens. Environ..

[bb0235] Okujeni A., van der Linden S., Suess S., Hostert P. (2017). Ensemble learning from synthetically mixed training data for quantifying urban land cover with support vector regression. IEEE J. Sel. Top. Appl. Earth Observations Remote Sensing.

[bb0240] Okujeni A., Canters F., Cooper S.D., Degerickx J., Heiden U., Hostert P. (2018). Generalizing machine learning regression models using multi-site spectral libraries for mapping vegetation-impervious-soil fractions across multiple cities. Remote Sens. Environ..

[bb0245] Olofsson P., Foody G.M., Herold M., Stehman S.V., Woodcock C.E., Wulder M.A. (2014). Good practices for estimating area and assessing accuracy of land change. Remote Sens. Environ..

[bb0250] Olson D.M., Dinerstein E., Wikramanayake E.D., Burgess N.D., Powell G.V.N., Underwood E.C. (2001). Terrestrial Ecoregions of the world. A new map of life on earth. BioScience.

[bb0255] Pesaresi M., Huadong G., Blaes X., Ehrlich D., Ferri S., Gueguen L. (2013). A global human settlement layer from optical HR/VHR RS data. Concept and first results. IEEE J. Sel. Top. Appl. Earth Observations Remote Sensing.

[bb0260] Pflugmacher D., Rabe A., Peters M., Hostert P. (2019). Mapping pan-European land cover using Landsat spectral-temporal metrics and the European LUCAS survey. Remote Sens. Environ..

[bb0265] Phinn S., Stanford M., Scarth P., Murray A.T., Shyy P.T. (2002). Monitoring the composition of urban environments based on the vegetation-impervious surface-soil (VIS) model by subpixel analysis techniques. Int. J. Remote Sens..

[bb0270] Powell R.L., Roberts D.A., Dennison P.E., Hess L. (2007). Sub-pixel mapping of urban land cover using multiple endmember spectral mixture analysis: Manaus, Brazil. Remote Sens. Environ..

[bb0275] Priem F., Okujeni A., van der Linden S., Frank Canters (2019). Comparing map-based and library-based training approaches for urban land-cover fraction mapping from Sentinel-2 imagery. Int. J. Appl. Earth Obs. Geoinf..

[bb0280] Reed B.C., Brown J.F., VanderZee D., Loveland T.R., Merchant J.W., Ohlen D.O. (1994). Measuring phenological variability from satellite imagery. J. Veg. Sci..

[bb0285] Ridd M.K. (1995). Exploring a V-I-S (vegetation-impervious surface-soil) model for urban ecosystem analysis through remote sensing. Comparative anatomy for cities. Int. J. Remote Sens..

[bb0290] Roberts D.A., Smith M.O., Adams J.B. (1993). Green vegetation, nonphotosynthetic vegetation, and soils in AVIRIS data. Remote Sens. Environ..

[bb0295] Roberts D.A., Gardner M., Church R., Ustin S., Scheer G., Green R.O. (1998). Mapping chaparral in the Santa Monica Mountains using multiple endmember spectral mixture models. Remote Sens. Environ..

[bb0300] Rosentreter J., Hagensieker R., Okujeni A., Roscher R., Wagner P.D., Waske B. (2017). Subpixel mapping of urban areas using EnMAP data and multioutput support vector regression. IEEE J. Sel. Top. Appl. Earth Observations Remote Sensing.

[bb0305] Schneider A. (2012). Monitoring land cover change in urban and peri-urban areas using dense time stacks of Landsat satellite data and a data mining approach. Remote Sens. Environ..

[bb0310] Schneider A., Friedl M.A., Potere D. (2010). Mapping global urban areas using MODIS 500-m data. New methods and datasets based on ‘urban ecoregions’. Remote Sens. Environ..

[bb0315] Schug F., Okujeni A., Hauer J., Hostert P., Nielsen J.Ø., van der Linden S. (2018). Mapping patterns of urban development in Ouagadougou, Burkina Faso, using machine learning regression modeling with bi-seasonal Landsat time series. Remote Sens. Environ..

[bb0320] Sexton J.O., Song X.-P., Huang C., Channan S., Baker M.E., Townshend J.R. (2013). Urban growth of the Washington, D.C.–Baltimore, MD metropolitan region from 1984 to 2010 by annual, Landsat-based estimates of impervious cover. Remote Sens. Environ..

[bb0325] Small C., Sousa D. (2016). Humans on earth. Global extents of anthropogenic land cover from remote sensing. Anthropocene.

[bb0330] Somers B., Asner G.P., Tits L., Coppin P. (2011). Endmember variability in spectral mixture analysis. A review. Remote Sens. Environ..

[bb0335] Song X.-P., Hansen M.C., Stehman S.V., Potapov P.V., Tyukavina A., Vermote E.F., Townshend J.R. (2018). Global land change from 1982 to 2016. Nature.

[bb0340] Stefanov W.L., Ramsey M.S., Christensen P.R. (2001). Monitoring urban land cover change. Remote Sens. Environ..

[bb0345] Stokes E.C., Seto K.C. (2019). Characterizing and measuring urban landscapes for sustainability. Environ. Res. Lett..

[bb0350] Suess S., van der Linden S., Okujeni A., Griffiths P., Leitão P.J., Schwieder M., Hostert P. (2018). Characterizing 32 years of shrub cover dynamics in southern Portugal using annual Landsat composites and machine learning regression modeling. Remote Sens. Environ..

[bb0355] Turner B.L., Lambin E.F., Reenberg A. (2007). The emergence of land change science for global environmental change and sustainability. Proc. Natl. Acad. Sci. U. S. A..

[bb0360] UNDESA (2018). World urbanization prospects. The 2018 Revision.

[bb0365] UNDESA (2019). World Population Prospects. The 2019 Revision.

[bb0370] United Nations (2015). World Urbanization Prospects. The 2014 Revision.

[bb0375] United Nations (2018). Sustainable Development Goals Report 2018.

[bb0380] USGS (2006). Shuttle Radar Topography Mission, 1 Arc Second.

[bb0385] van der Linden S., Okujeni A., Canters F., Degerickx J., Heiden U., Hostert P. (2018). Imaging spectroscopy of urban environments. Surv. Geophys..

[bb0390] Verrelst J., Muñoz J., Alonso L., Delegido J., Riveira J.P., Camps-Valls G., Moreno J. (2012). Machine learning regression algorithms for biophysical parameter retrieval. Opportunities for Sentinel-2 and -3. Remote Sens. Environ..

[bb0395] Walton J.T. (2008). Subpixel urban land cover estimation. Photogramm Eng Remote Sensing.

[bb0400] Weng Q. (2012). Remote sensing of impervious surfaces in the urban areas. Requirements, methods, and trends. Remote Sens. Environ..

[bb0405] Wentz E., York A.M., Alberti M., Conrow L., Fischer H., Inostroza L. (2018). Six fundamental aspects for conceptualizing multidimensional urban form. A spatial mapping perspective. Landsc. Urban Plan..

[bb0410] Wetherley E.B., McFadden J.P., Roberts D.A. (2018). Megacity-scale analysis of urban vegetation temperatures. Remote Sens. Environ..

[bb0415] Wu C., Murray A.T. (2003). Estimating impervious surface distribution by spectral mixture analysis. Remote Sens. Environ..

[bb0420] Yang J., Li P. (2015). Impervious surface extraction in urban areas from high spatial resolution imagery using linear spectral unmixing. Remote Sensing Applications: Society and Environment.

[bb0425] Yuan F., Wu C., Bauer M.E. (2008). Comparison of spectral analysis techniques for impervious surface estimation using Landsat imagery. photogramm eng remote sensing.

